# Plasma Cardiac Troponin-I Concentration in Normal Horses and in Horses with Cardiac Abnormalities

**DOI:** 10.3390/ani15010092

**Published:** 2025-01-03

**Authors:** Jonathan H. Foreman, Brett S. Tennent-Brown, Mark A. Oyama, D. David Sisson

**Affiliations:** Department of Veterinary Clinical Medicine, College of Veterinary Medicine, University of Illinois, 1008 West Hazelwood Drive, Urbana, IL 61802, USA

**Keywords:** equine, ventricular arrhythmia, cardiomyopathy, myocarditis, cardiac muscle injury

## Abstract

Cardiac troponin-I (cTnI) is a highly sensitive and specific marker of myocardial injury detectable in plasma by immunoassay techniques. Inclusion criteria over a 3-year period required a diagnosis of cardiac disease accompanied by electrocardiographic (ECG) and cardiac ultrasound examinations (n = 23) in adult horses (≥2 years of age). A second group of normal adult ponies (n = 12) was studied as a reference group. Heparinized jugular venous blood samples were collected and centrifuged within 30 min, and the plasma was separated and frozen at −70 °C for subsequent batched cTnI analysis. The lower limit of detection was 0.01 ng/mL, and the upper limit was 100 ng/mL of plasma. Normal equine plasma cTnI concentrations ranged from 0.01 to 0.03 ng/mL (n = 12). Horses with non-arrhythmogenic murmurs (n = 4) included tricuspid (0.05 ng/mL cTnI), mitral (0.07), and aortic insufficiencies (0.01, 0.02). Horses with benign atrial fibrillation (n = 8) had a cTnI range of <0.01–0.09 ng/mL, with four horses having cTnI concentrations falling slightly outside the reference range (0.04, 0.05, 0.06, and 0.09). Horses with ventricular arrhythmias (ventricular premature contractions or ventricular tachycardia) and documentable myocardial toxicities or immunological reactions (n = 5) had cTnI concentrations of 0.05, 0.21, 0.31, 15.18, and >100 ng/mL. Horses with ventricular arrhythmias but no documentation of myocardial toxicity (n = 3) had cTnI concentrations of 0.34, 0.46, and 80.42 ng/mL. When grouped by arrhythmia type and compared using the Mann–Whitney Rank Sum Test, the median ventricular arrhythmia cTnI (0.40 ng/mL) was significantly higher than the median atrial fibrillation cTnI (0.04 ng/mL, *p* < 0.001). It was concluded that horses with myocardial toxicities and ventricular arrhythmias often had severe elevations in plasma cTnI.

## Simple Summary

Cardiac troponin-I (cTnI) in plasma is used daily as a biomarker indicative of the severity of myocardial damage in humans suffering cardiac ischemia due to coronary artery disease, commonly referred to as “heart attacks”. Because troponin is so similar in humans and domestic animal species, human immunoassays are often used to measure cTnI concentrations in domestic animals. All assays should, however, have normal reference ranges specific to the type of assay and the species of concern. We used a human chemiluminescence assay to measure cTnI concentrations in normal ponies, to establish an assay-specific reference range, and in adult horses with clinical cardiac disease confirmed by electrocardiography and ultrasonography. Horses with atrial fibrillation sometimes had mild increases in cTnI, with concentrations outside the reference range. Horses with ventricular arrhythmias had a 10-fold higher median cTnI than horses with atrial fibrillation, confirming the utility of cTnI plasma measurements in horses with suspected ventricular premature contractions or ventricular tachycardia. Ventricular arrhythmias carry a poorer prognosis for survival than atrial fibrillation in horses.

## 1. Introduction

Cardiac muscle injury can lead to the release of myocardial proteins into general circulation. These proteins may then be measured in blood or plasma as biomarkers indicative of myocardial injury [1,2,3]. Cardiac troponin and the myocardial band isoform of creatine kinase (CKMB) are the most common proteins used as indicators of myocardial injury in humans [1,2,3]. Troponin molecules normally serve in a regulatory capacity in the cardiac actin/myosin complex, but myocardial damage can result in the release of troponin into circulation and thus provide an opportunity for measurement as a biomarker of cardiac injury.

Different subunit forms of the troponin protein can be measured in plasma. They are designated as cardiac troponin-I (cTnI) and cardiac troponin-T (cTnT), with the cardiac isoforms having been shown to be antigenically distinct from the skeletal muscle isoforms [4,5,6]. A third troponin (cTnC) is shared by both cardiac and skeletal muscle tissue and thus is not considered to be as myocardial-specific as cTnI and cTnT [1]. Troponin-I is inhibitory to the actin and myosin interaction until calcium is bound by cTnC [7]. Cardiac troponin-I is normally bound to actin by cTnT, but sarcomere injury can result in detachment and release into the cytosol and extracellular space and eventually into circulation [7].

Cardiac troponin-I and -T exist intracellularly in a one-to-one ratio but are detected differently once released from damaged myocardium [8]. Kinetically, the plasma cTnI concentration is detectable earlier (from 2 to 6 h), peaks higher (between 18 and 24 h), and decrements faster than cTnT, with a plasma half-life in humans of approximately 2 h [1]. Cardiac troponin-I is, therefore, considered to be a highly sensitive and specific biomarker of myocardial injury and can be detected in plasma by various immunoassay techniques. In humans suffering from myocardial ischemic injury due to coronary artery obstruction, troponin concentrations in plasma are often used for diagnosis, monitoring of damage progression, and prognosis. Sensitivity has been increased in human myocardial infarction patients with the development of high-sensitive troponin (hs-cTn) assays [3].

In veterinary medicine, the use of human immunoassays for the detection of plasma cTnI concentrations has been reported previously in dogs [7,9], cats [9], cattle [10], and horses [11,12,13,14]. The close homology of troponin isoforms between human and domestic animal species permits the use of human immunoassays for the measurement of troponin in dogs and horses [7]. However, troponin quantification in horses should be considered assay-specific [2]. In particular, reported ranges of normal values vary between assays by as much as an order of magnitude depending on the assay used. In this study, we hypothesized that horses with clinically apparent cardiac disease would have elevated plasma cTnI when compared to normal horse plasma concentrations, with cTnI concentrations measured using a human immunoassay that has been reported on sparingly in previous studies with horses.

## 2. Materials and Methods

Inclusion criteria. Horses presented to the University of Illinois Veterinary Teaching Hospital for possible cardiac problems underwent complete physical examinations, including thorough auscultation. When cardiac abnormalities were suspected based on auscultation findings, the use of subsequent procedures was approved by the Institutional Animal Care and Use Committee. Horses were examined by electrocardiography and ultrasonography. The inclusion criteria for this series of cases over a 3-year period required a diagnosis of cardiac disease in an adult horse (≥2 years of age) accompanied by electrocardiographic (ECG) and cardiac ultrasound examinations (n = 23 cardiac cases). A second group of normal adult ponies (n = 12) was also studied as a reference group.

Samples. Jugular venous blood samples were collected at rest, without any preceding exercise, from normal and cardiac patients in 8 mL evacuated blood tubes containing 143 IU of sodium heparin. The samples were not collected in any consistent relationship to prior meals; cardiac cases were spontaneous clinical cases sampled as horses arrived in the hospital or after one or more nights of hospitalization. The samples were centrifuged within 30 min of collection, and the plasma was separated and frozen at −70 °C for subsequent batched cTnI analysis.

Analysis. Cardiac TnI analysis was performed with the human Access AccuTnI assay (Beckman Coulter, Inc., Fullerton, CA, USA) [1]. The AccuTnI assay uses a chemiluminescent sandwich ELISA technique. The linearity of this assay has been validated previously in horse plasma samples [14], but reports of the use of this assay in spontaneous clinical equine cases are limited [7,15]. The lower limit of detection in our study was 0.01 ng/mL, and the upper limit was 100 ng/mL of equine plasma. The results from normal ponies were used to establish a reference range to compare results from horses with cardiac diagnoses.

Statistical Analysis. Mean values ± standard deviations and the median plasma cTnI concentrations were calculated for normal ponies and the two groups of horses with either atrial fibrillation or ventricular arrhythmias. Cardiac troponin-I results were compared for atrial versus ventricular groups using Student’s *t*-test with a significance level set at *p* < 0.05. The Mann–Whitney Rank Sum Test was used if the data were not normally distributed.

## 3. Results

Diagnosis (disease, ECG, and ultrasound), breed, age, disposition (alive to discharge, dead, or euthanized while hospitalized), and the results of plasma cTnI concentrations for normal ponies and horses with cardiac disease are summarized in Table 1. Normal equine plasma cTnI concentrations ranged from 0.01 to 0.03 ng/mL (n = 12) with a mean of 0.015 ± 0.007 and a median of 0.01 ng/mL. Horses with auscultable murmurs (n = 4) and no arrhythmia included tricuspid insufficiency (0.05 ng/mL), mitral insufficiency (0.07 ng/mL), and aortic insufficiency (0.01 and 0.02 ng/mL). Horses with benign atrial fibrillation (n = 8) had cTnI ranges of <0.01–0.09 ng/mL, with four horses having cTnI concentrations falling slightly outside the reference range (0.04, 0.05, 0.06, and 0.09 ng/mL).

Horses with ventricular arrhythmias (ventricular premature contractions (VPCs) and/or ventricular tachycardia) and documentable myocardial toxicities or immunological reactions (n = 5) had cTnI concentrations of 0.09, 0.21, 0.31, 15.18, and >100 ng/mL (Table 1). The causes included Streptococcal myositis (n = 3), white snakeroot poisoning (n = 1), and red maple leaf poisoning (n = 1). Horses with ventricular arrhythmias with no known cause (n = 4) had cTnI concentrations of 0.12, 0.34, 0.46, and 80.42 ng/mL. One 15-year-old American Saddlebred mare with a cTnI concentration of 80.42 ng/mL presented with an HR of 120/min, auscultable arrhythmia, depression, and anorexia. On ECG, she manifested VPCs, which progressed rapidly to ventricular tachycardia. With intravenous fluids and a CRI of lidocaine, she converted to a normal sinus rhythm within 12 h of treatment onset. In 40- and 60-day follow-up examinations, she had cTnI concentrations of 0.35 and 0.10 ng/mL plasma, respectively.

Horses with Streptococcal myositis had a history of exposure to strangles, respiratory signs (nasal exudate, lymph node enlargement), elevated plasma creatine kinase (CK) and aspartate transaminase (AST) activities, and elevated cTnI concentrations (Table 1). Two Quarter Horses from the same stable developed clinical disease contemporaneously and the third was unrelated to the former two. One horse with white snakeroot poisoning had a history of exposure to white snakeroot, profound weakness, elevated plasma CK and AST, and an elevated cTnI concentration (Table 1). One horse with red maple toxicity had elevated cTnI concentration (Table 1) after a storm knocked down red maple trees in the pasture. One broodmare diagnosed with a peripartum uterine ligament hemorrhage, severe blood loss, weakness, anemia, and ventricular arrhythmias had a plasma cTnI concentration >100 ng/mL (Table 1).

The mean cTnI values for atrial and ventricular groups were 0.04 ± 0.03 (range < 0.01–0.09) and 29.71 ± 44.56 ng/mL (range 0.09–>100), respectively. However, because the data were not normally distributed, the more appropriate statistical comparison was made using median values, with ventricular arrhythmia horses having a median cTnI 10-fold higher (0.40 ng/mL) than atrial arrhythmia horses (0.04 ng/mL) (*p* < 0.001).

## 4. Discussion

In the absence of cardiac disease, cTnI is tightly bound to actin via cTnT within the cardiac actin-tropomyosin complex, and plasma cTnI concentrations remain low to undetectable. Our reference range (0.01–0.03 ng/mL) was identical to that described by Nath et al. [12,13] using a different assay in 18 Thoroughbred (TB) horses. Using the same assay as we used here in our horses, Kraus et al. described a slightly wider reference range (0.01–0.06 ng/mL), but 95% of their samples fell within our narrower range [16]. All but one of the 135 horses (67 Standardbred (STB), 34 TB, and 35 Warmblood (WB)) tested by Nostel and Häggström, using an enzyme immunoassay, had cTnI concentrations <0.022 ug/L (same as ng/mL) [17]. Using our assay, Van Der Vekens et al. opined that a value of 0.035 ng/mL should be considered the high normal for horses, with values above considered indicative of cardiac disease [15]. Using a human colorimetric immunoassay, Phillips et al. considered a wider range of values to represent normal in 20 TB horses (0–0.35 ng/mL), with a mean normal value of 0.047 ng/mL [11]. It is imperative that interpretations of patient cTnI concentrations be made in comparison to assay-specific normal values or reference ranges [2]. Elevations in plasma cTnI concentrations above the specific reference range have been associated with myocardial damage in humans [1,3,4], dogs [7], and horses [12,13]. In our series of cases, benign atrial arrhythmias occasionally resulted in slight increases in cTnI to concentrations outside the reference range, but ventricular arrhythmias were more often associated with much larger cTnI increases (Table 1).

Elevations in plasma cTnI concentrations in dogs have been detected in various pathologies and can, therefore, be used validly as blood-based biomarkers of cardiac disease [7]. In studies using two different cTnI assays, median normal plasma values in dogs have been reported to be 0.02 ng/mL (n = 41 dogs) [9] and 0.03 ng/mL (n = 176, range 0.01–0.23 ng/mL) [7]. Clearly, cTnI is conserved intracellularly in normal dogs, just as it was in our normal ponies. However, using the same assay as our study, plasma cTnI was shown to increase significantly with age, even in normal dogs [7]. Elevations of cTnI have been reported in dogs with cardiomyopathy (median 0.14 ng/mL, range 0.03–1.88, n = 26), mitral valve disease (median 0.11, range 0.01–9.53, n = 37), and subaortic stenosis (median 0.08, range 0.01–0.94, n = 30) [7]. In dogs with cardiomyopathy, those dogs with cTnI concentrations greater than 0.20 ng/mL had significantly shorter survival times (median 112 days) than those with cTnI concentrations less than 0.20 ng/mL (median 357 days), regardless of the censoring status of dogs which were euthanized [7]. In dogs, cTnI has proven to be a valuable biomarker indicative of cardiac muscle injury [7].

Myocardial damage caused by dietary toxins or acute anemia was often indicated by the highest concentrations of plasma cTnI measured in our series of horses. Experimental or clinical exposure to skeletal muscle toxins and/or myocardial-specific toxins in horses has been reported to result in measurable increases in plasma cTnI [18]. White snakeroot (*Ageratina altissima*, previously *Eupatorium rugosum*) causes skeletal muscle degeneration and necrosis, particularly in the myocardium [19], resulting in tachycardia, arrhythmia, and profound weakness as seen in our case with a cTnI > 100 ng/mL. The ionophore antibiotics monensin and lasalocid are used as growth-promoting agents in cattle but are profoundly toxic to the horse myocardium. Exposures are accidental in horses, either by the use of treated cattle feed or, more often, by feeding with horse feed contaminated by ionophores in the milling process. Both monensin [16,20] and lasalocid [21,22] exposure have been associated with increases in plasma cTnI concentrations in horses.

Other myocardial toxins have been reported to result in elevations of equine cTnI, including cantharidin [23], rattlesnake venom (*Crotalus* spp.) [24], avocado (*Persea americana*) [25], and rayless goldenrod (*Isocoma pluriflora* [26]. Iatrogenic increases in cTnI concentrations have resulted from the administration of cobalt [27] and dobutamine [28,29,30], but not clenbuterol [31], levothyroxine [32], tildipirosin [33], or various methods of general anesthesia [34]. Interestingly, fumonisin is a known depressor of cardiovascular function, but Smith et al. reported normal cTnI concentrations in horses with experimentally induced fumonisin toxicity [35]. This cardiovascular depression in the absence of evidence of myocardial damage (normal cTnI) indirectly supports the suspicion that fumonisin toxicity is caused by a blockade of L-type Ca^++^ channels rather than direct myocardial injury [35,36].

Increases in plasma cardiac troponin have been documented after challenges with various infectious agents. Strangles (*Streptococcus equi* subspecies *equi*) has been associated clinically with the development of myositis [37] and increased plasma cTnI [38], as in three of the cardiac cases in our series (Table 1). Neither an equine influenza virus challenge in 23 vaccinated ponies [39] nor vaccination against equine herpesvirus-1 in 18 horses [40] resulted in a change in cTnI concentrations in plasma. Piroplasmosis in horses has been associated with increases in plasma cTnI [41,42], likely due to severe anemia and presumed myocardial hypoxemia that may accompany acute presentation [43].

In this study, horses with simple acquired murmurs had low cTnI concentrations (0.07 ng/mL or less), similar to a report by Traschel et al. [44] on six Warmblood horses with mitral insufficiency. Benign atrial fibrillation resulted in measurable but minimal increases in cTnI in our series, as previously described by Nath et al. [12]. Several studies have documented that transvenous electrical cardiac conversion (TVEC) for the treatment of atrial fibrillation has not been associated with subsequently increased cTnI, indicating that TVEC does not cause measurable myocardial injury [45,46,47]. However, ventricular arrhythmias were associated with much higher values for cTnI in our horses, with the lowest being 0.09 ng/mL and with two horses measuring beyond the upper limit of detection of the assay (>100 ng/mL). The median cTnI for ventricular arrhythmias was 10-fold higher than the atrial fibrillation cTnI median (*p* < 0.001). This finding is in keeping with that of others where simple murmurs or atrial fibrillation resulted in minimal but measurable increases in cTnI, but ventricular arrhythmias resulted in elevations of cTnI of much greater magnitude [12,48]. Interestingly, in our series, the TB broodmare with uterine artery hemorrhage (Table 1) illustrates that the severe loss of oxygen-carrying capacity (via hemorrhage causing acute anemia) can cause sufficient presumed myocardial damage to result in large elevations in plasma cTnI. Despite the severity of her cTnI increase beyond the upper limit of detection, this mare survived by intensive treatment with intravenous fluids and a CRI of lidocaine. The relationship between acute hemorrhage and the development of ventricular arrhythmias in horses has been described previously by Navas de Solis et al. [49].

One horse in this series survived to discharge and then had two follow-up examinations that included repeated cTnI measurements. The massive decrease from her initial cTnI of 80.42 down to 0.35 and 0.10 ng/mL demonstrates the value of longitudinal measurements of cTnI in the course of treatment and follow-up of equine cardiac cases. While the cause of her ventricular arrhythmias was never documented, the use of cTnI to diagnose the severity of her presentation, followed by her response to treatment and recovery, demonstrates the utility of cTnI monitoring. Interestingly and significantly, however, her cTnI had still not returned fully to the normal range by 60 days after her presentation. Large elevations in cTnI in our cases were not always associated with a poor prognosis, but this outcome is undoubtedly influenced by motivated clinicians and owners. All four of the deaths in our series were from euthanasia of ventricular arrhythmia cases, not from spontaneous death; however, that choice by owners can be attributed to a myriad of factors, including finances, a poor prognostic picture painted by the clinicians, and/or a reduced utility of the horse in the owner’s plans. The progression of cases in Table 1 makes it clear, however, that the prognosis decreased for ventricular arrhythmias compared to atrial arrhythmias or uncomplicated murmurs.

The effect of exercise stress on cTnI concentration is often raised as a potential confounding variable on cTnI utility in horses and warrants discussion. Plasma cTnI concentrations have been investigated in horses at pasture, during training, and after various types of strenuous exercise. Measurements have been reported in normal pastured and race-trained Thoroughbreds [11,50], race-trained Standardbreds [51,52,53,54,55], and Norwegian–Swedish trotters [46]. Thoroughbred horses performing in fast-paced races pulling chuckwagons in Canada had increased post-exercise hs-cTnT [56], but draft horses contesting in much slower-paced heavier weight pulling contests did not [57]. Endurance exercise has been documented to cause increases in cTnI [58,59,60,61], but such increases were not associated definitively with horses failing to finish races. Eventing cross-country (CC) exercise resulted in increased cTnI concentrations in 40 out of 55 horses examined (73%), and these increases persisted in 18 of the 20 horses still available for sampling (90%) on the morning after CC [62]. One report for jumpers showed mild cTnI elevations after a single course of jumps [63]. Our horses’ cTnI concentrations were measured at rest without a confounding bout of exercise.

The limitations of this study are ascribed to the limited number of complete cases meeting all the inclusion criteria. The relationship between cardiotoxin exposure, myocardial injury, and elevated plasma cTnI concentration is clear historically but not statistically proven here due to the limited number of those cases. Follow-up samples could be used in the future to assess the relationship between plasma cTnI and clinical improvement or decline. Longitudinal assessment of cTnI as clinical cases progress over time should provide valuable data to aid in the determination of response to therapy and prognosis [2].

## 5. Conclusions

It was concluded that horses with myocardial toxicities or acute anemia and ventricular arrhythmias often had demonstrable elevations in plasma cTnI well outside the reference range we established for this assay. Horses with more benign conditions, such as uncomplicated valvular insufficiencies or atrial fibrillation, had normal to minor elevations of cTnI falling slightly outside the reference range, but cTnI was not nearly as elevated as it was in horses with ventricular arrhythmias.

## Figures and Tables

**Table 1:** Summary of plasma cardiac troponin I (cTnI) concentrations from normal ponies (n = 12) and abnormal horses (n = 23), and the associated breed, age, clinical diagnosis, electrocardiographic (ECG) diagnosis, and ultrasound diagnosis. All four dead horses were euthanized with the owner’s request and consent. Breed abbreviations: TB = Thoroughbred, STB = Standardbred, WB = warmblood, QH = Quarter Horse, ASB = American Saddlebred. Westph = Westphalian. ECG abbreviations: VPC = Ventricular Premature Contraction, VT = Ventricular Tachycardia.

Plasma cTnI (ng/mL)	Status	Ultrasound Diagnosis	ECG Diagnosis	Age (yrs)	Breed	Diagnosis
Range 0.01–0.03 Mean 0.015 ± 0.007 Median 0.01	Alive	Normal	Normal	Estimated 2–6	Pony	Normal (n = 12)
0.07	Alive	Mitral insufficiency	Normal	26	Trakehner	Acquired murmur (1)
0.05	Alive	Tricuspid insufficiency	Normal	5	TB	Acquired murmur (1)
0.01	Alive	Aortic insufficiency	Normal	Unknown	TB	Acquired murmur (3)
0.02	Alive	Aortic insufficiency	Normal	15	STB	
0.06	Alive	Aortic insufficiency	Normal	17	QH	
<0.01	Alive	Normal	Atrial fibrillation	13	TB	Atrial fibrillation (8)
0.01	Alive	Normal	Atrial fibrillation	15	STB	
0.02	Alive	Normal	Atrial fibrillation	13	Westph	
0.02	Alive	Normal	Atrial fibrillation	7	STB	
0.04	Alive	Normal	Atrial fibrillation	10	Clydesdale	
0.05	Alive	Normal	Atrial fibrillation	13	STB	
0.06	Alive	Normal	Atrial fibrillation	21	WB	
0.09	Alive	Normal	Atrial fibrillation	15	STB	
0.09	Alive	Normal	VPCs	7	QH	Streptococcal myositis (3)
0.31	Dead	Normal	VPCs	2	QH	
15.18	Alive	Normal	VPCs	2	Arabian	
0.12	Dead	Normal	VT	12	TB	Unknown cause (4)
0.34	Alive	Normal	VPCs, VT	19	Mixed	
0.46	Dead	Normal	VT	20	QH	
80.42, 0.35 at 40 days after discharge, 0.10 at 60 days after discharge	Alive	Normal	VPCs, VT	15	ASB	
0.21	Alive	Normal	VPCs	15	QH	Red maple leaf toxicity (1)
>100	Dead	Normal	VT	6	QH	White snakeroot toxicity (1)
>100	Alive	Normal	VT	Unknown	TB	Uterine artery hemorrhage (1)

## Data Availability

Data are contained within the article.
